# Age-dependent VDR peak DNA methylation as a mechanism for latitude-dependent multiple sclerosis risk

**DOI:** 10.1186/s13072-021-00383-x

**Published:** 2021-02-04

**Authors:** Lawrence T. C. Ong, Stephen D. Schibeci, Nicole L. Fewings, David R. Booth, Grant P. Parnell

**Affiliations:** 1 Centre for Immunology and Allergy Research, Westmead Institute for Medical Research, The University of Sydney, 176 Hawkesbury Rd, Westmead, NSW 2145 Australia; 2 Department of Immunology, Westmead Hospital, Cnr Darcy and Hawkesbury Rds, Westmead, NSW 2145 Australia

**Keywords:** DNA methylation, Calcitriol, Epigenetics, Vitamin D, Myeloid, VDR binding site

## Abstract

**Background:**

The mechanisms linking UV radiation and vitamin D exposure to the risk of acquiring the latitude and critical period-dependent autoimmune disease, multiple sclerosis, is unclear. We examined the effect of vitamin D on DNA methylation and DNA methylation at vitamin D receptor binding sites in adult and paediatric myeloid cells. This was accomplished through differentiating CD34+ haematopoietic progenitors into CD14+ mononuclear phagocytes, in the presence and absence of calcitriol.

**Results:**

Few DNA methylation changes occurred in cells treated with calcitriol. However, several VDR-binding sites demonstrated increased DNA methylation in cells of adult origin when compared to cells of paediatric origin. This phenomenon was not observed at other transcription factor binding sites. Genes associated with these sites were enriched for intracellular signalling and cell activation pathways involved in myeloid cell differentiation and adaptive immune system regulation.

**Conclusion:**

These results suggest vitamin D exposure at critical periods during development may contribute to latitude-related differences in autoimmune disease incidence.

## Background

The prevalence of autoimmune diseases such as multiple sclerosis (MS), type 1 diabetes mellitus, rheumatoid arthritis and atopic diseases such as asthma, follow a latitude gradient, with increasing prevalence at latitudes more distant from the equator [1, 2]. Ultraviolet light exposure and hence serum vitamin D levels are known to correlate with latitude, yet our understanding of the mechanisms linking vitamin D to immune disease remains incomplete [3–6]. DNA methylation, an important epigenetic mark, has been posited as a potential link between environmental exposures and disease due to its susceptibility to environmental change [7] and relative stability over time [8].

Some latitude-dependent diseases such as MS also demonstrate a critical period, where risk factors such as latitude of residence appear to exert their influence during childhood and adolescence [9–11]. This critical period is perhaps underpinned by age-related susceptibility to alterations in DNA methylation. DNA methylation changes have been detected in leukocyte development at key histone modifiers, chromatin remodellers and immune susceptibility loci within the first 5 years of life [12]. DNA methylation changes proceed more rapidly in normal childhood development, with changes in peripheral blood occurring at a 3- to 4-fold higher rate compared with adults [13]. Prenatal susceptibility to environmental insults such as famine, are also highly influenced by gestational age, resulting in persistent DNA methylation changes into adulthood [14, 15].

Vitamin D exerts its genomic effects through binding the vitamin D receptor (VDR). Binding of the active form of vitamin D, calcitriol, results in heterodimerisation of the VDR with the retinoid X receptor (RXR). This heterodimer binds regions of DNA known as vitamin D response elements (VDREs), which lie in the promoter regions of vitamin D responsive genes and lead to subsequent upregulation or suppression of DNA transcription. DNA methylation at VDREs may therefore interfere with the effects of calcitriol on transcriptional regulation.

Whilst the genomic effects of vitamin D have been well characterised, its effects on DNA methylation are poorly understood. A study of cholecalciferol supplementation and mouse CD4+ T cells in experimental autoimmune encephalomyelitis (EAE; a mouse model of MS), showed global decreases in DNA methylation. This was associated with changes in the expression of enzymes involved in the establishment and maintenance of DNA methylation marks, with concomitant decreases in CD4 + T cell proliferation and differentiation into inflammatory Th1 and Th17 subsets [16]. Another study found increases in Helios + Foxp3 + T regulatory cells with 1,25(OH)_2_vitamin D_3_ (calcitriol) supplementation that were associated with amelioration of EAE, with an increase rather than decrease in global DNA methylation [17].

More MS risk genes are predominantly expressed in mononuclear phagocytic cells than any other cell subset [18]. These cells are likely to be important in the pathogenesis of MS through their regulation of immune cell differentiation, via mechanisms such as antigen presentation and expression of key vitamin D-associated MS risk genes [18, 19]. An epigenome-wide study of vitamin D treatment on the human monocyte cell line, THP-1, found marked changes in chromatin accessibility due to vitamin D with maximal chromatin opening after 24 h [20]. Despite this, ex vivo mononuclear cells cultured with vitamin D for up to 120 h did not show any differentially methylated CpGs, despite extensive changes in gene expression [21]. The authors suggested donor age may have affected DNA methylation plasticity, however other factors including duration of culture, use of terminally differentiated cells and heterogeneous cell population, may also have contributed to the apparent lack of effect on DNA methylation.

This study therefore hypothesised that differentiating haematopoietic progenitors into monocyte/macrophage lineage cells in the presence of calcitriol would result in age-dependent DNA methylation changes. Thus, we sought to determine whether immune cell DNA methylation is affected by exposure to calcitriol. Secondly, because the multiple sclerosis latitude gradient appears to be mediated by a critical period, we sought to determine whether calcitriol-related DNA methylation changes vary with age. Because the genomic effects of vitamin D are mediated by its receptor, we also examined whether DNA methylation changes at corresponding binding sites are age dependent.

## Results

### Calcitriol results in changes in cell number, morphology and phenotype in cell culture

**Fig. 1:**
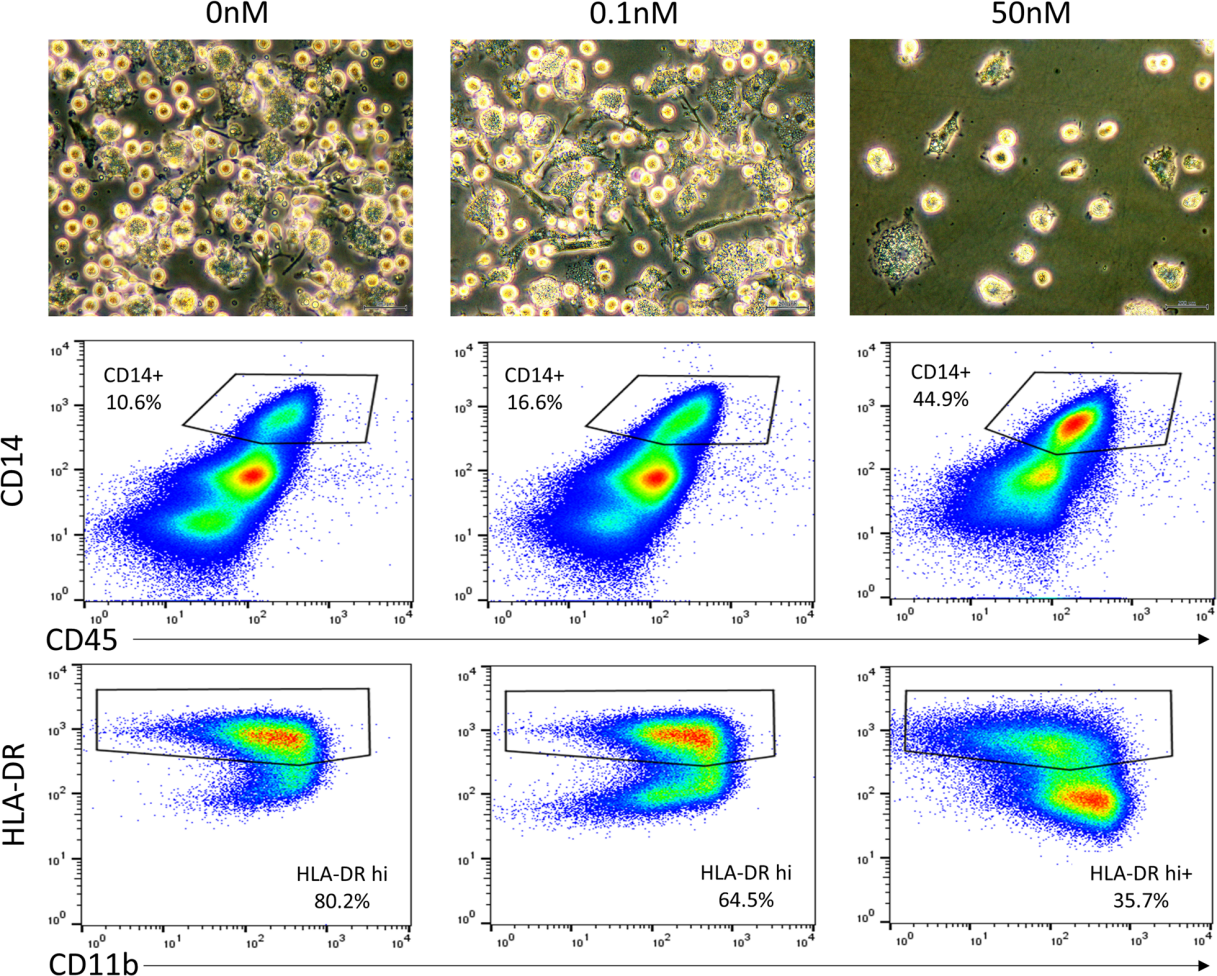
Light microscopy and flow cytometric characterisation of cultured cells at day 22. Cultured cells of adult origin with media containing 0 nM calcitriol (left), 0.1 nM calcitriol (centre) and 50 nM calcitriol (right). There were marked morphologic and immunophenotypic changes, with overall decrease in cell number, fewer fusiform-shaped cells, greater CD14+ proportion and decreases in HLA-DR and CD16 expression (not shown) at higher calcitriol concentrations

We conducted preliminary cell culture with varying calcitriol concentrations to determine effects on cell morphology and immunophenotype. Using CD34+ haematopoietic progenitor cells originating from an adult subject, at day 22, we noted a marked decrease in cell number and a less activated immunophenotype with higher concentrations of calcitriol (Fig. 1). This preliminary experiment confirmed that a physiological concentration of calcitriol (0.1 nM) was sufficient to elicit phenotypic changes in cultured cells. In the final experiment that cultured cells from two adult and two paediatric subjects, overall CD14+ percentage as a subset of CD45+ cells, was greater in cells of paediatric origin than those of adult origin with mean CD14+ proportion 78.9% vs 11.7% (*p* < 4.99 × 10^−4^, two-tailed t-test).

### DNA methylation varies more due to age and individual differences than due to calcitriol

**Fig. 2:**
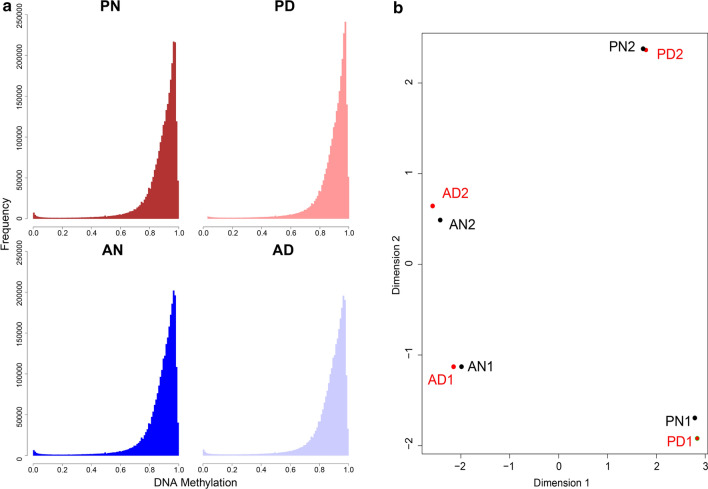
Global DNA methylation by age and calcitriol status. **a** Frequency histograms of 1-kb tile, genome-wide DNA methylation, showing similar distribution of DNA methylation between conditions. **b** Multidimensional scaling analysis of CpG-wise methylation values demonstrating only minor differences in DNA methylation with the addition of calcitriol. A: adult, P: paediatric, N: no calcitriol, D: with calcitriol, 1 or 2 refer to adult or paediatric subject 1 or 2

Whole-genome bisulfite sequencing reads, alignment statistics, bisulfite conversion rates and coverage rates are detailed in Additional file 1. On average, 96% of genome-wide CpGs were covered at a depth of 16×. Genome -wide DNA methylation did not vary to a large extent by age or calcitriol exposure. The average proportion of methylated reads was 0.83 for each of the four sample categories (adult ± vitamin D, paediatric ± vitamin D; see Fig. 2a). Multidimensional scaling analysis of methylation values by sample found little difference in DNA methylation secondary to calcitriol. Differences due to calcitriol exposure were generally much smaller than those due to individual differences or age (Fig. 2b).

There were few individual CpGs that differed in methylation state following calcitriol exposure. In cells of adult origin, there were 502 differentially methylated CpGs (FDR < 0.05) corresponding to 29 autosomal and 11 mitochondrial genes/gene promoters. Amongst cells of paediatric origin, there were 37 differentially methylated CpGs corresponding to 2 genes. None of the differentially methylated CpGs overlapped between adult and paediatric samples or with VDR peaks. Of all adult differentially methylated CpGs, a subset mapped to the promoter region of one MS risk gene, PAPD7. Details of differentially methylated CpGs can be found in Additional files 2 and 3.

### DNA methylation varies markedly by donor age at myeloid VDR peaks

**Fig. 3:**
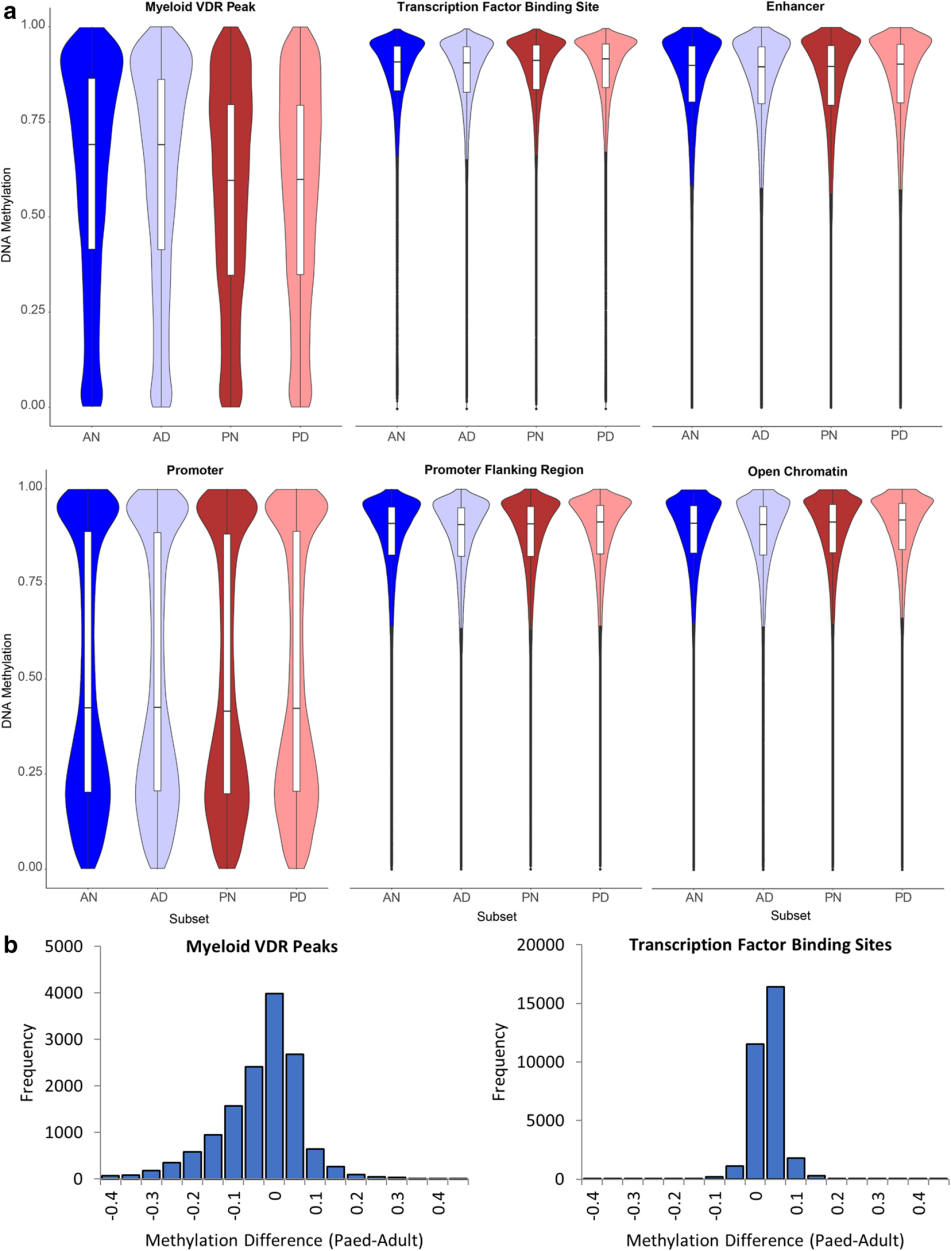
Distribution of DNA methylation by genomic feature. **a** Violin plots of DNA methylation across various genomic features by cell origin and culture condition. Myeloid VDR peaks demonstrated DNA methylation that was skewed towards lower methylation levels in cells of paediatric origin in comparison to those of adult origin. The effects of calcitriol on DNA methylation distribution was not evident. **b** Methylation difference between cells of paediatric and adult origin at myeloid VDR peaks and transcription factor binding sites, showing skewing towards paediatric hypomethylation at myeloid VDR peaks, but not at transcription factor binding sites. A: adult, P: paediatric, N: no calcitriol, D: with calcitriol, 1 or 2 refer to adult or paediatric subject 1 or 2

**Fig. 4:**
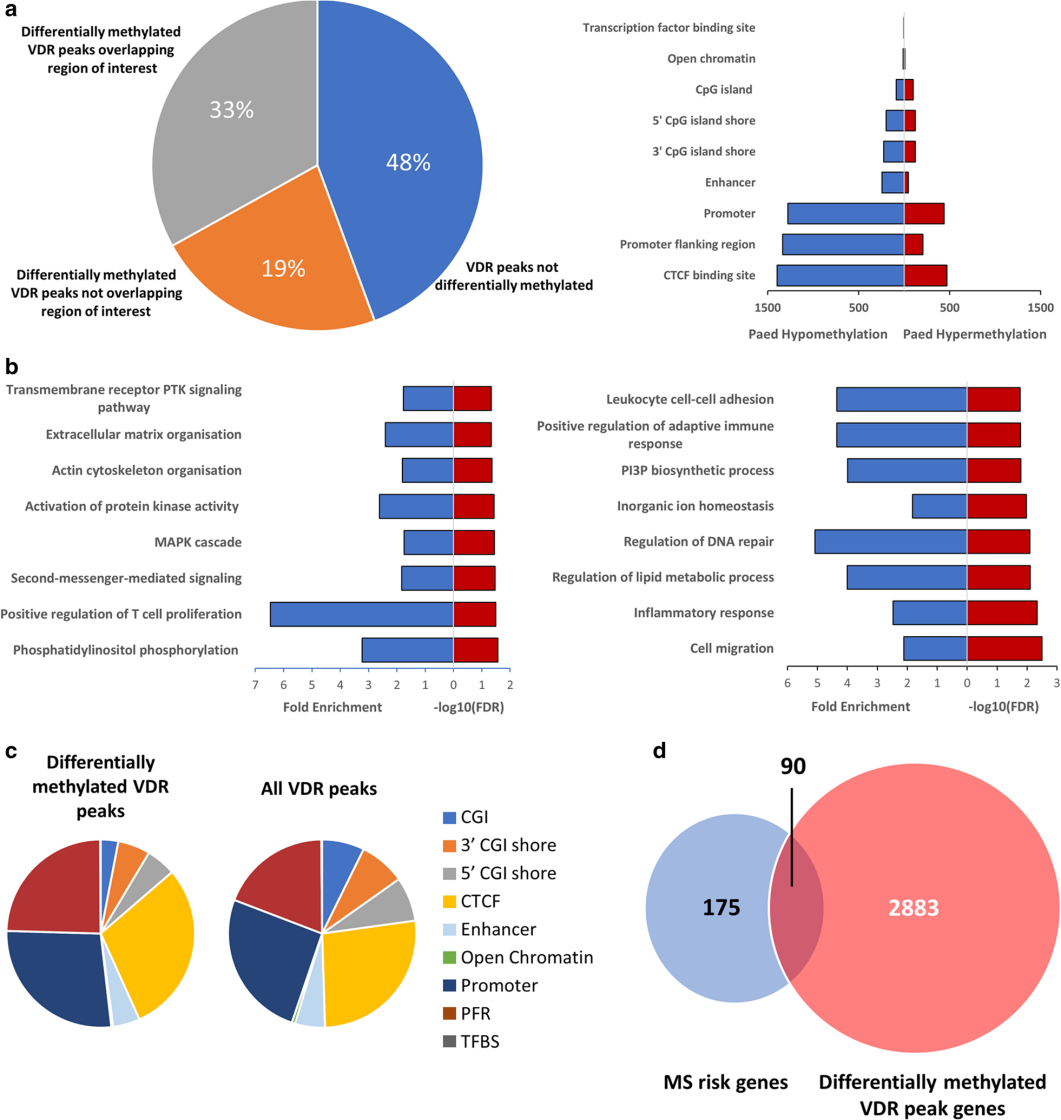
Characteristics of differentially methylated VDR peaks. **a** Breakdown of VDR peaks based on differential methylation status and overlap with regions of interest (regulatory regions, CpG islands and island shores; left) and differentially methylated VDR peaks overlapping with regions of interest (right). The majority of regulatory regions demonstrated hypomethylation in cells of paediatric origin. **b** Overrepresented GO terms (FDR < 0.05) associated with differentially methylated VDR peaks. **c** Breakdown of differentially methylated myeloid VDR peaks and corresponding annotation overlaps compared with all annotated VDR peaks. **d** Overlap of currently known non-HLA MS risk genes and their overlap with differentially methylated myeloid VDR peaks (Additional file 5). PTK: protein tyrosine kinase, PI3P: phosphatidylinositol-3-phosphate, CGI: CpG island, PFR: promoter flanking region, TFBS: transcription factor binding site

VDR binding sites are another mechanism by which calcitriol may exert effects on gene expression. There were marked differences in the distribution of DNA methylation between samples of adult and paediatric origin at myeloid VDR peaks, which were not apparent at other transcription factor binding sites or regulatory regions (Fig. 3). There was overall lower DNA methylation at myeloid VDR peaks in cells of paediatric origin (*p* < 2.2 × 10^−16^, Wilcoxon rank sum test). RADmeth [22] was used to call differentially methylated CpGs between samples of adult and paediatric origin regardless of exposure to calcitriol. There were 26,134 differentially methylated CpGs corresponding to 7244 VDR peaks (52% of all myeloid VDR peaks) and 2973 genes (Additional file 4). In comparison, analysis of CD14+ transcription factor binding sites (TFBS) yielded 7125 differentially methylated CpGs corresponding to 1896 TFBS (22% of annotated CD14+ TFBS). In comparison to TFBS, differential methylation was proportionally greater at VDR peaks than TFBS (χ^2^ = 3983, *p* < 1 × 10^−5^). A statistical overrepresentation test [23] (Panther GO-slim annotation version 14.1, released March 12, 2019) found many immune and intracellular signalling ontologies to be enriched amongst genes corresponding to differentially methylated myeloid VDR peaks (Fig. 4b) (Additional file 5).

The genomic annotations overlapping differentially methylated myeloid VDR peaks were then determined. Of interest, were CD14+ Ensembl regulatory build annotations and hg19 CpG island/shore annotations. Five prime and 3′ CpG island shores were designated as 2000-bp upstream and downstream of the corresponding hg19 CpG island, respectively. Fifty-two percent of myeloid VDR peaks contained differentially methylated CpGs, with most of these being hypomethylated in cells of paediatric origin relative to cells of adult origin. The peaks overlapped predominantly with promoter regions, promoter flanking regions and CTCF binding sites, although not in the expected proportion in comparison to all myeloid VDR peaks (χ^2^ = 300.8, *p* < 2.2 × 10^−16^, df = 8), suggestive of enrichment for specific genomic annotations (Fig. 4c). The overlap of 352 CpGs previously identified as markers of biological age [24] with differentially methylated VDR peaks was ascertained to determine whether differential methylation could be attributed to the cumulative effects of epigenetic maintenance. None of the differentially methylated VDR peaks contained a “clock” CpG. Ninety of the differentially methylated VDR peaks overlapped with non-HLA multiple sclerosis risk genes (Fig. 4d), underlining the potential importance of DNA methylation differences in this latitude-dependent autoimmune disease.

### Transcriptomic effects of calcitriol vary by age

**Fig. 5:**
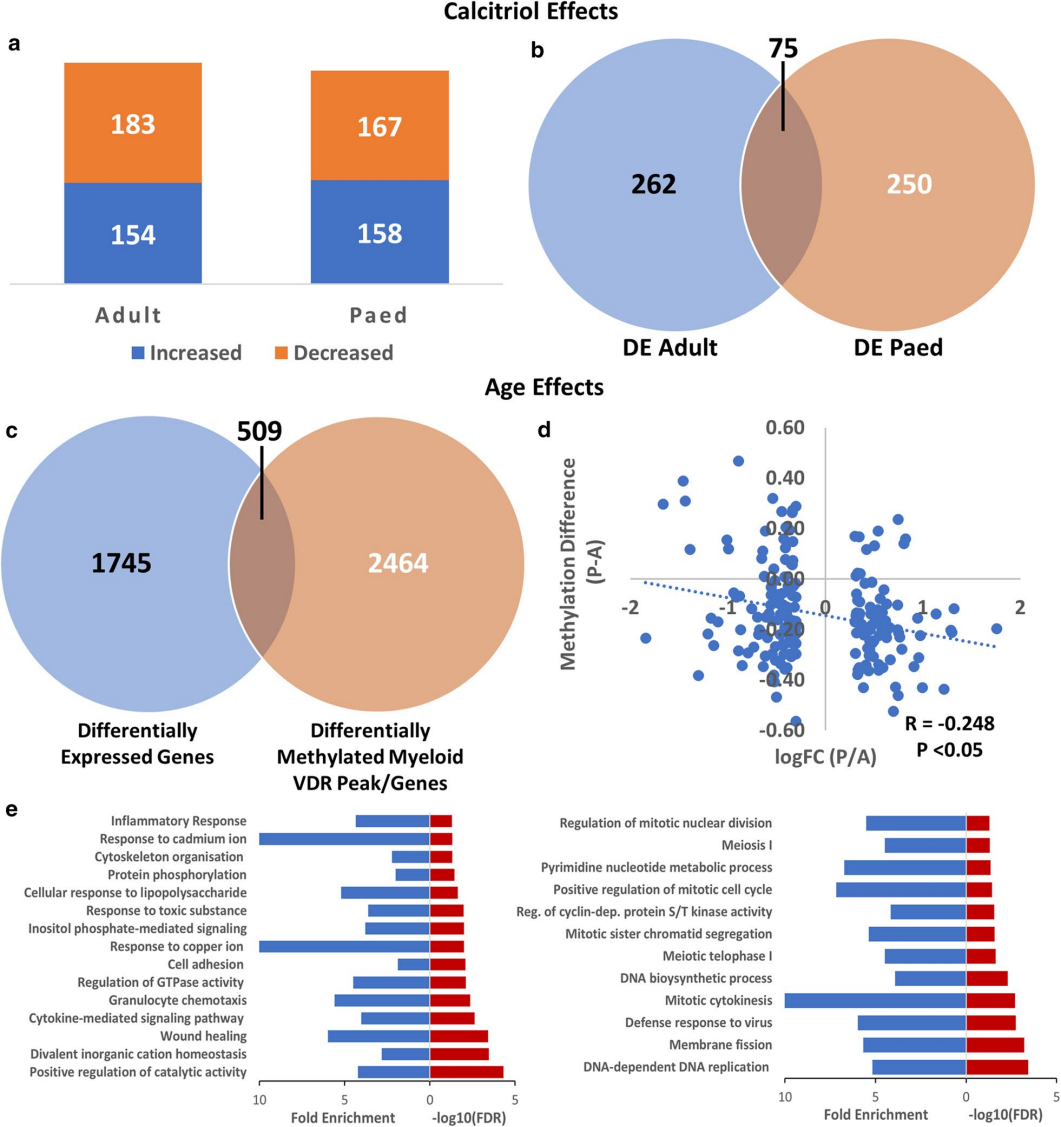
Differentially expressed genes, associated gene ontologies and DNA methylation overlap. **a** Number of genes demonstrating changes in expression (> twofold) due to calcitriol in cells of adult and paediatric origin. **b** A minority of common genes are differentially expressed in response to calcitriol amongst cells of adult and paediatric origin. **c** Overlap between differentially expressed genes and differentially methylated myeloid VDR peaks/genes when comparing cells of adult and paediatric origin. **d** Scatter plot of overlapping sites from **c** corresponding to annotated promoter regions. There was a significant negative correlation between methylation difference (paediatric–adult) and log fold-change (paediatric/adult). **e** Overrepresented GO biological process terms (FDR < 0.05)

The alignment rate for RNA-seq reads to the hg19 genome ranged from 84.4 to 90.0%. Transcriptomic analysis by RNA-seq identified 183 downregulated and 154 upregulated genes amongst adult cells treated with calcitriol compared to no calcitriol, using a fold-change threshold of two. Amongst cells of paediatric origin, there were 167 downregulated and 158 upregulated genes due to calcitriol (Fig. 5a). Overall, only 75 differentially expressed genes overlapped between cells of adult and paediatric origin (Fig. 5b), 57 in the same direction with calcitriol exposure and 18 in opposite directions (Additional file 6). A statistical overrepresentation test did not yield any statistically significant GO terms associated with any of the differentially expressed gene sets. None of the differentially expressed genes secondary to calcitriol were differentially methylated.

### Age-dependent transcriptomic effects are greater than calcitriol-dependent effects

**Fig. 6:**
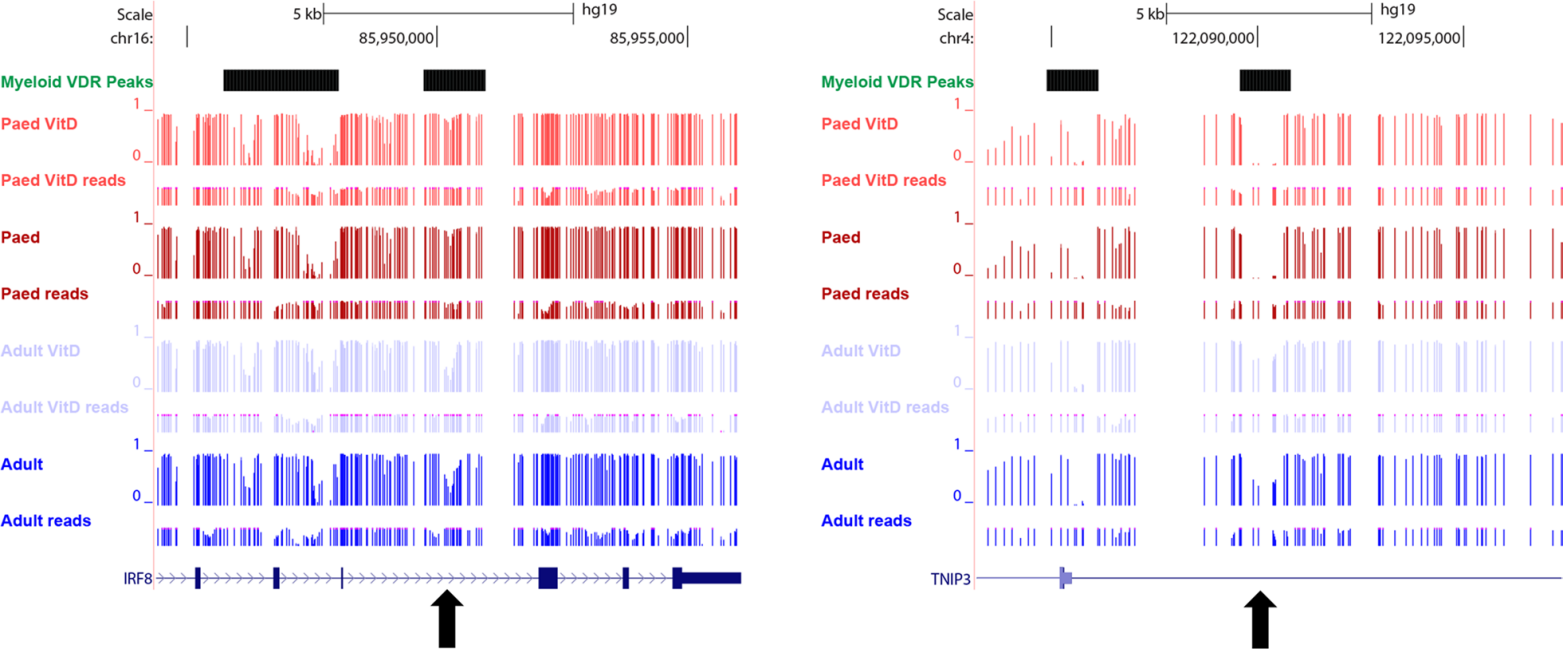
An example of differential methylation at myeloid VDR peaks overlapping with MS risk genes. In cells of paediatric origin, DNA methylation was increased at IRF8 (left) and decreased at TNIP3 (right) relative to cells of adult origin. Both genes were also differentially expressed between cells of adult and paediatric origin (see Additional file 7). Black arrows denote differentially methylated regions. Red tracks: paediatric, blue tracks: adult

A greater number of differentially expressed genes were observed between adult and paediatric cells, independent of calcitriol exposure. We found 1002 genes were downregulated and 1252 upregulated in cells of paediatric compared to adult origin. Of these genes, 509 overlapped with differentially methylated myeloid VDR peaks (p < 9.38 × 10^−6^, hypergeometric test; Figs. 5c and 6; co-location with genomic annotations is noted in Additional file 7). There was a negative correlation between expression fold-change and methylation difference at CD14+ annotated promoters, consistent with the known relationship between DNA methylation at promoter regions and gene expression (Fig. 5d). Of the differentially expressed genes, those underexpressed in cells of paediatric origin were enriched for biological processes relating to inflammation, intracellular signalling and metal ion homeostasis. Those overexpressed in cells of paediatric origin were associated with cellular replication and cell cycle processes (Fig. 5e).

Of the 509 overlapping differentially expressed/methylated genes, 28 overlapped with 265 non-HLA MS risk genes [25] (*p* = 1.82 × 10^−6^, hypergeometric test). Two hundred and seventy-six of these overlapping genes underexpressed in paediatric cells were enriched for the GO terms “cell surface receptor signalling”, “cell migration”, “intracellular signal transduction” and “protein phosphorylation”. The remaining 233 overexpressed genes were not enriched for any biological process terms (see Additional file 7).

## Discussion

This study examined potential interactions between calcitriol and DNA methylation in myeloid cells, to identify mechanisms underlying critical periods in the development of latitude-dependent autoimmune diseases such as MS. Calcitriol addition resulted in marked morphologic and phenotypic effects, however, DNA methylation changes were relatively minor in comparison. Vitamin D-independent DNA methylation changes differed between cells of paediatric and adult origin, especially at myeloid VDR peaks. MS risk genes were prominent among differentially methylated VDR peaks. Gene expression changes due to calcitriol, differed markedly between adult and paediatric cells, with only ~ 22% of differentially expressed genes common to both conditions. The changes in VDR peak methylation due to age may be sufficient to drive the profoundly different transcriptomes between paediatric and adult derived mononuclear phagocytic cells, with evidence of MS risk gene involvement.

The calcitriol-bound VDR-RXR complex binds to VDREs and participates in transcriptional regulation. Therefore, DNA methylation changes at these regions are likely to have important functional consequences. We found 52% of myeloid VDR peaks were differentially methylated between cells of adult and paediatric origin. Differential methylation was enriched above background CD14+ TFBS, providing support for VDR specificity of age-dependent changes. Most of the differentially methylated peaks also displayed decreased methylation in cells of paediatric origin. At 17% of these sites, there was concomitant differential gene expression, suggesting an immediate functional effect of these methylation differences within a subset of myeloid VDR peaks. Biological processes associated with underexpressed genes in paediatric cells were predominantly associated with inflammation, intracellular signalling and response to divalent cations, whereas those associated with overexpressed genes were predominantly associated with cellular proliferation.

Genes in *cis* with most differentially methylated VDR peaks were associated with biological processes important in inflammation and cellular differentiation, but not with changes in gene expression. VDR binding sites proximal to genes encoding PI3K subunits (including PIK3R1/3/6, PIK3CG, PIK3C2B) were differentially methylated between cells of adult and paediatric origin. The PI3K molecular pathway plays a role in myeloid cell differentiation [27, 28], monocyte antimycobacterial activity [29] and promotion of macrophage differentiation [30]. Genes relating to the MAPK cascade were also enriched amongst differentially methylated VDR peak genes. The MAPK cascade is involved in significant crosstalk with the PI3K/AKT pathway [31] and has pleiotropic effects in monocytes/macrophages depending on the triggering stimulus and cell type. These effects include differentiation and activation [32]. Together, the differential methylation of genes relating to both the PI3K and MAPK pathways suggest that the differing potential for vitamin D-related myeloid cell differentiation between adult and paediatric cells may be encoded by DNA methylation.

Genes relating to the regulation of the adaptive immune response and T cell proliferation were also differentially methylated between adult and paediatric cells. This suggests that myeloid cells are differentially primed to influence the adaptive immune system in childhood compared with adulthood.

Consistent with previous work on vitamin D supplementation in mononuclear cells [21], DNA methylation in our myeloid cells appeared to be relatively insensitive to the effects of calcitriol. This occurred despite differentiation from haematopoietic progenitor cells from paediatric donors, which would presumably demonstrate greater DNA methylation plasticity in response to environmental stimuli. In contrast, the previously documented effect of vitamin D on DNA methylation in mouse CD4+ T cells was much more prominent [16]. Many effects of vitamin D on human monocytes/macrophages may be mediated by epigenetic marks other than DNA methylation [20].

**Fig. 7:**
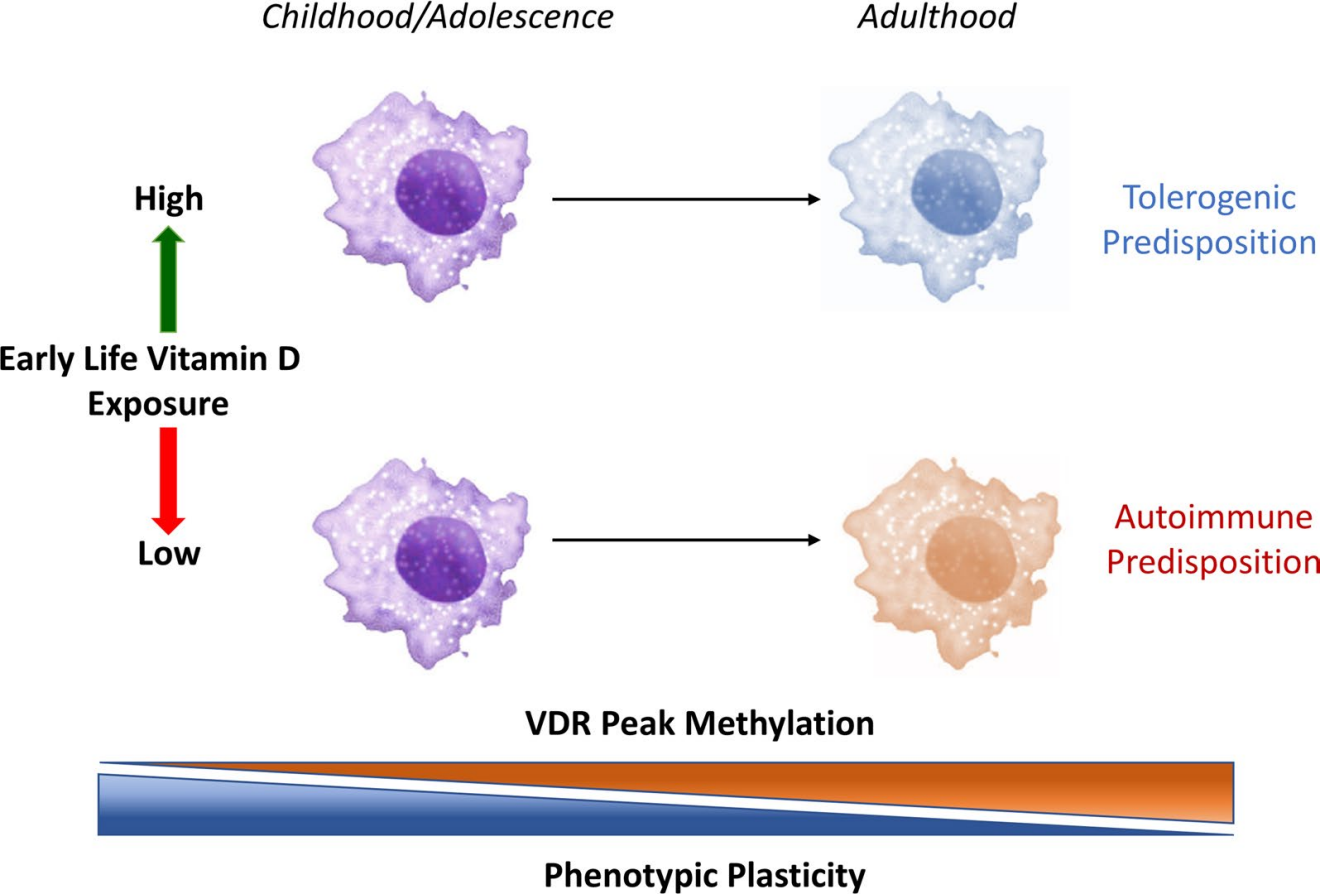
A potential mechanism for the development of autoimmune disease risk dependent on early life vitamin D exposure. Decreased VDR binding site methylation in early life increases phenotypic plasticity and susceptibility to vitamin D exposure. Because tissue macrophages persist for months to years, phenotypic settings resulting from vitamin D exposure in early life may lead to a tolerogenic or autoimmune propensity in later life. Macrophage images adapted from [26]

How age-dependent methylomic differences at myeloid VDR binding sites confer long-term risk for latitude-dependent diseases is unclear. Monocytes typically persist in the circulation for up to ~ 1 week [33], and would be an unlikely substrate for DNA methylation-dependent risk unless they migrate to peripheral sites. These tissue resident macrophages are known to persist for much longer periods (months to years) [34], perhaps transmitting early life influenced phenotypic changes that either predispose or limit propensity to autoimmunity in later life (Fig. 7). Another possibility is that VDR agonism (or lack thereof) during early life leads to persistent changes in VDR binding site methylation and later susceptibility to VDR effector activities. Therefore, this study has potentially important implications for the prevention of latitude-dependent autoimmune diseases. It suggests that a therapeutic window exists where vitamin D agonism may be effective in altering disease risk through modulation of myeloid cell development and function.

One potential reason for finding minimal DNA methylation changes with vitamin D could be related to the duration of the cell cultures. Increasing passage number is associated with increases in DNA methylation [24], potentially distorting or obscuring the effects of vitamin D or age on DNA methylation. Due to the lack of CD14+ cells at earlier stages of culture, we were also unable to determine temporal effects on DNA methylation with vitamin D exposure. It has been previously shown that chromatin accessibility due to calcitriol exposure peaks at 24 h and virtually returns to baseline levels after 48 h [20]. It could be argued however, that DNA methylation changes are less likely to occur within these time frames in comparison to histone modifications and non-coding RNAs. Finally, whether the phenomenon of age-related differential methylation at VDR binding sites occurs in vivo requires further investigation.

Future studies will need to ascertain the robustness of our present findings across a greater number of biological replicates. This study also raises questions regarding age-dependent VDR methylation in other cell lineages as well as haematopoietic progenitor cells, and whether VDR methylation settings might be transmitted to progeny cells. The model of altered tissue macrophage phenotype might also be amenable to study by comparison of their phenotype/function in MS with normal individuals, for example in co-culture.

## Conclusions

Whilst vitamin D has minor effects on the myeloid methylome, age-dependent differences in VDR peak DNA methylation suggest vitamin D exposure at critical periods in immune system development may contribute to well-characterised latitude-related differences in autoimmune disease risk.

## Methods

### Cell isolation

Adult peripheral blood was extracted by injecting approximately 50 ml of Dulbecco’s PBS (DPBS) with 10% acid citrate dextrose (Sigma Aldrich) into leukocyte reduction system (LRS) chambers discarded following plateletpheresis from two male subjects and allowing the chamber to drain under gravity. Umbilical cord blood units obtained from one male and one female donor, were diluted in a 1:1 ratio with DPBS. Mononuclear cells from both adult and paediatric samples were then obtained by density gradient centrifugation (Ficoll-Paque PLUS, GE Healthcare). Positive selection of CD34+ haematopoietic progenitor cells was then performed using CD34 MicroBead Kit Ultrapure Microbeads (Miltenyi Biotec) at a volume of 50 µl per 10^8^ cells, with an autoMACS Pro Separator (Miltenyi Biotec) as per manufacturer’s instructions. Purified cells were plated at a density of 5 × 10^4^ cells/ml of media.

### Cell culture

CD14+ cells were cultured from haematopoietic cell precursors (CD34+) using a previously published protocol with minor modifications [35]. The culture cocktail contained X-VIVO10 (Lonza), Albumex (Seqirus) 0.05%, SCF (Peprotech) 200 ng/ml, GM-CSF (Peprotech) 0.03ug/ml, M-CSF (premium grade; Miltenyi Biotec) 5000U/ml, IL-6 (Peprotech) 10 ng/ml, FLT3 ligand (Peprotech) 50 ng/ml and gentamicin (Sigma Aldrich) 50 µg/ml. Calcitriol (1,25(OH)_2_vitamin D_3_; BioGems) was added at a physiological concentration of 0.1 nM. Cells were incubated at 37 °C with 5% CO_2_ for 1 week before replating at a density of 1 × 10^5^ cells/ml of media. Media was then changed every third day by demi-depletion. Cells were harvested on day 21.

Harvested cells were centrifuged at 300×*g* for 5 min before the supernatant was removed. The cells were resuspended in 1 ml of chilled PBS, stained with 1 µl of LIVE/DEAD Fixable Aqua Dead Cell Stain (Life Technologies) and incubated on ice for 30 min. The cells were then washed and stained with the following antibody cocktail: CD45 BUV395 (BD Horizon), CD14 BV421 (BD), CD16 BV650 (BD), HLA-DR FITC (BD Pharmingen), CD34 PE (BD Pharmingen), CD11b APC (BD). FACS sorting was carried out on a BD Influx and CD14+ cells subjected to two washes with DPBS at 1000×*g* before storage at − 80 °C.

### Whole-genome bisulfite sequencing

DNA was extracted using QIAamp DNA extraction kit (Qiagen) as per manufacturer’s instructions. Whole-genome bisulfite sequencing libraries were generated with the Accel-NGS Methyl-seq DNA Library Kit (Swift Biosciences) and sequenced on a HiSeq X10 (Illumina) in 150 bp PE mode with PhiX spike-into counteract low sequence diversity.

### Data QC, alignment and processing

The quality of raw sequences was ascertained using FastQC [36]. Quality trimming was carried out using Trim galore [37] in paired end mode with the following parameters -quality 20, –three_prime_clip_R1 5, –clip_R2 15. Reads were aligned to the hg19 genome using the Wildcard Alignment Tool (WALT) with default settings and the output.sam files converted to.mr files using *to*-*mr* before further processing with Methpipe [38]. Firstly, duplicate removal was performed using *duplicate*-*remover*, followed by estimation of bisulfite conversion rates and coverage/methylation level statistics using *bsrate* and *levels,* respectively. Methylation calls were made using *methcounts* (using the -n option for CpG context cytosines only) before *symmetric*-*cpgs* was used to extract and merge methylation data from both strands. Regional methylation analysis was performed using the *roimethstat* module (with -P and -v options), to determine methylation state within a prespecified region of interest.

### Differential methylation analysis

RADmeth [22] was utilised for differential methylation analysis. The effects of calcitriol and age were considered separately by comparing the effects of calcitriol amongst cells of adult and paediatric origin separately. To further examine the specific effects of vitamin D, myeloid vitamin D receptor (VDR) peaks [39] and a 500 bp region up and downstream were also examined. DNA methylation at these sites was compared to experimentally validated CD14+ transcription factor binding sites (TFBS) [40] (± 500 bp). Overlap between differentially methylated regions and genes/genomic annotations was determined using Bedtools [41] *intersect*, with *closest* being used to determine the nearest gene to differentially methylated myeloid VDR peaks.

### RNA sequencing

Due to low cell number, RNA-seq was not performed on cells cultured from one of the adult subjects. RNA was extracted from other cultured CD14+ cells with the RNeasy Mini Kit (Qiagen) as per manufacturer’s instructions. Sequencing libraries were generated with the QIAseq Stranded mRNA Select Kit (Qiagen) and sequenced on the Novaseq 6000 (Illumina) using 100 bp SE mode. The quality of raw sequences was ascertained using FastQC [36]. Fifteen base pairs were trimmed from the start of each read using Trimmomatic [42] before alignment with TopHat2 [43]. Assignment of aligned reads to genes was performed using featureCounts [44]. Quantile normalised RPKM values were utilised to determine fold-change differences. Genes with aggregate read counts < 100 or belonging to sex chromosomes were excluded from further analysis, resulting in 12,492 genes. Only non-zero numerators or denominators were kept for fold-change and downstream calculations.

## Supplementary Information

**Additional file 1.** Cell harvest, WGBS alignment, bisulfite conversion and coverage statistics.

**Additional file 2.** Genome-wide paediatric differentially methylated CpGs.

**Additional file 3.** Genome-wide adult differentially methylated CpGs.

**Additional file 4.** Differentially methylated VDR myeloid peaks.

**Additional file 5.** MS risk genes overlapping differentially methylated VDR peaks.

**Additional file 6.** Differentially expressed genes, vitamin D vs no vitamin D.

**Additional file 7.** Differentially expressed genes, adult vs paediatric.

## Data Availability

The datasets used and/or analysed during the current study are available from the corresponding author on reasonable request.
